# InAs/InAsSb Strained-Layer Superlattice Mid-Wavelength Infrared Detector for High-Temperature Operation

**DOI:** 10.3390/mi10120806

**Published:** 2019-11-22

**Authors:** Gamini Ariyawansa, Joshua Duran, Charles Reyner, John Scheihing

**Affiliations:** Air Force Research Laboratory, Sensors Directorate, Wright-Patterson Air Force Base, OH 45433, USA; joshua.duran.2@us.af.mil (J.D.); charles.reyner.1@us.af.mil (C.R.); john.scheihing@us.af.mil (J.S.)

**Keywords:** Infrared detector, strained layer superlattice, InAs/InAsSb, absorption coefficient, barrier detector, high operating temperature

## Abstract

This paper reports an InAs/InAsSb strained-layer superlattice (SLS) mid-wavelength infrared detector and a focal plane array particularly suited for high-temperature operation. Utilizing the *n*B*n* architecture, the detector structure was grown by molecular beam epitaxy and consists of a 5.5 µm thick *n*-type SLS as the infrared-absorbing element. Through detailed characterization, it was found that the detector exhibits a cut-off wavelength of 5.5 um, a peak external quantum efficiency (without anti-reflection coating) of 56%, and a dark current of 3.4 × 10^−4^ A/cm^2^, which is a factor of 9 times Rule 07, at 160 K temperature. It was also found that the quantum efficiency increases with temperature and reaches ~56% at 140 K, which is probably due to the diffusion length being shorter than the absorber thickness at temperatures below 140 K. A 320 × 256 focal plane array was also fabricated and tested, revealing noise equivalent temperature difference of ~10 mK at 80 K with f/2.3 optics and 3 ms integration time. The overall performance indicates that these SLS detectors have the potential to reach the performance comparable to InSb detectors at temperatures higher than 80 K, enabling high-temperature operation.

## 1. Introduction

Lower cost, size, weight, and power (C-SWaP) have become a requirement for many infrared imaging systems. A great impact on C-SWaP could be achieved through high operating-temperature (HOT) [1] sensors and focal plane arrays (FPAs), which in turn require developing suitable sensor materials exhibiting high uniformity, high stability, and good electrical and optical properties. One class of materials that has the potential to do just that are III–V, antimony-based, strained-layer superlattices [2] (SLSs), which have already shown impressive results. HOT [1,3] capability has been the primary goal in the mid-wavelength infrared (MWIR) band and a few demonstrations [4] have been already reported. Research groups have explored different detector architectures (*n*B*n* [5], *n*B*p* [6], *p*B*p* [7], *X*B*n* [8], CBIRDs [9] etc.) and pixel geometries [5,10] across a multitude of SLS designs [11,12,13,14,15,16] to mitigate generation-recombination (G-R) current and surface-leakage current, while maximizing electrical/optical properties. Among those, detectors with InAs/InAsSb SLSs incorporated in the *n*B*n* architecture have received special attention due to high carrier lifetime [16,17,18] and reduced complexity of SLS growth [4]. Since the first demonstration of SLSs for infrared (IR) detectors [12], InAs/Ga(In)Sb SLSs continue to improve [19], while InGaAs/InAsSb SLSs [15,20] have also shown promising results. Researchers are currently addressing the poor hole mobility [21] and carrier localization [22,23] effects in *n*-type SLS to improve the diffusion length. They have also explored *p*-type SLS detectors, but surface passivation leading to surface leakage current [24,25] remains an insurmountable problem. In this paper, the focus is on a detector utilizing *n*-type InAs/InAsSb SLSs based on the *n*B*n* architecture, with the emphasis on HOT capability. Detector design, material characterization, and detector performance are discussed in detail, while FPA fabrication and testing are briefly discussed. 

## 2. Strained-Layer Superlattice (SLS) Design and Detector Structure 

**Figure 1:**
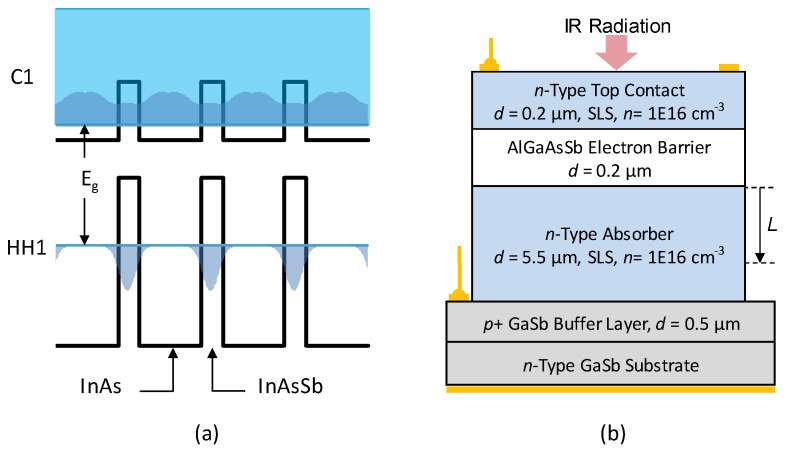
(**a**) InAs/InGaAs strained-layer superlattice (SLS) design showing the conduction and valence band profile for the bulk material as well as the valence and conduction minibands of the superlattice. The superlattice bandgap is also indicated as *E_g_*. (**b**) Structure of the *n*B*n* detector consisting of SLS layers as the absorber and contact layers and a bulk AlGaAsSb layer as the electron barrier.

Group III–V antimony-based SLSs are periodic structures of thin layers of semiconductor materials typically grown on GaSb substrates, which comprise a band-engineered artificial infrared material. In the InAs/InAsSb SLS reported here, the unit cell consists of 16 ML InAs and 6 ML InAsSb layers and the total unit cell thickness was then adjusted to achieve the desired bandgap and spectral response. The band structure of the superlattice was calculated using NRLMultiband software [26] and the band parameters such as the bandgap and carrier effective masses as well as material properties such as absorption coefficient were obtained. Figure 1a illustrates the conduction band (CB) and valence band (VB) profile of the bulk constituents of the superlattice along with superlattice minibands (HH1 and C1) and its bandgap (*E_g_*). The electron and hole probability distributions are also shown, indicating nearly free electrons in the C1 miniband and heavily confined holes in the HH1 miniband. This is a typical feature of InAs/InAsSb SLSs and one can optimize the design [15] to maximize the electron and hole wave function overlap in order to maximize the absorption coefficient and vertical hole mobility. The designed value of *E_g_* is 234 meV (5.3 µm) at 80 K in order to cover the entire 3–5 µm atmospheric band (MWIR). 

The SLS shown in Figure 1a was incorporated into an *n*B*n* architecture in order to build a detector. Figure 1b shows the complete structure of the *n*B*n* detector grown on GaSb substrate by molecular beam epitaxy at a commercial foundry. The active elements in the structure include a 5.5 µm thick *n*-type SLS absorber, a 0.2 µm thick AlGaAsSb electron barrier layer, and a 0.2 µm thick *n*-type SLS top contact layer. For single element device characterization, mesas were fabricated using standard photolithography, wet chemical etching, and metallization processes. A fully fabricated mesa device is illustrated in Figure 1b. In addition, a metallic mirror was also deposited on the backside of the wafer; this mirror provides a double pass optical geometry under front-side illumination, which approximates the performance of a backside-illuminated FPA. 

## 3. Characteristics of Detectors

**Figure 2:**
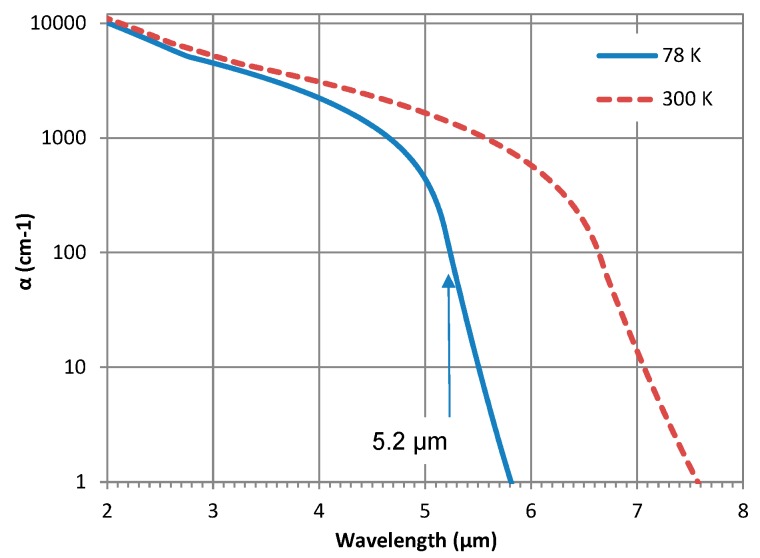
Measured absorption coefficient spectra of the InAs/InAsSb SLS at 78 and 300 K.

Material and device characterization was performed at a range of temperatures from 78 to 300 K. The absorption coefficient (α) was determined from transmission and reflection measurements using a Fourier transform infrared (FTIR) spectrometer; the details of the method are discussed elsewhere [27]. The absorption coefficient spectra for the superlattice reported here is shown in Figure 2 at 78 and 300 K. Also indicated in the Figure 2 is the cut-off wavelength (~5.2 µm) corresponding to the inflection point of the spectrum (also the edge of the Urbach [28] tail). This value is very close to the designed bandgap of the superlattice (5.3 µm). The value of α at 4 µm and 300 K is 3081 cm^−1^ and the average α in the 3–5 µm band is 3461 cm^−1^. Furthermore, the cut-off wavelength shifts to about 6.5 µm at 300 K, as expected. These spectra were used for calculating the absorption efficiency in the absorber for comparison against the measured quantum efficiency (QE) of the detector, which will be discussed later.

**Figure 3:**
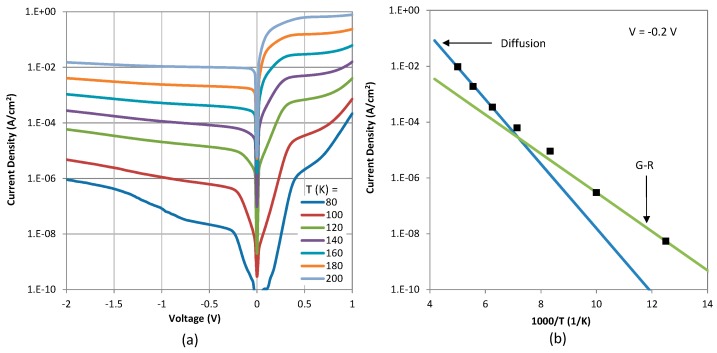
(**a**) Dark current-voltage-temperature (IVT) characteristics of a 400 × 400 µm size *n*B*n* detector and (**b**) Arrhenius plot at a bias voltage of –0.2 V. The linear fit to the current at T > 160 K yields an activation energy of 203 meV.

The fully processed detectors were packaged in leadless chip carriers and wire-bonded to the chip carrier leads to make electrical contacts. Dark current and photocurrent measurements were carried out after mounting the packaged devices in a liquid nitrogen pour-filled dewar. The dark current-voltage-temperature (IVT) characteristics measured in the 80–240 K temperature range are shown in Figure 3a. Here, the bias polarity is defined as negative (positive) when a negative (positive) voltage is applied on the top contact. The *n*B*n* detector is operated under negative bias where majority electrons flowing from the top contact to the bottom contact are blocked by the electron barrier, while photo-generated minority holes are collected at the top contact. Figure 3b shows the variation of the dark current density (*J_d_*) under –0.2 V bias with temperature (*T*) with a linear fitting to the experimental data at temperatures higher than 160 K. Based on the slope of *J_d_*/*T*^3^ vs. 1/*T* plot, the activation energy was calculated to be approximately 203 meV. This value is very close to the bandgap of the SLS at ~200 K (confirmed by the spectral cut-off discussed later). It also confirms that the dark current at T > 160 K is diffusion limited. At lower temperatures, the dark current of SLS-based *n*B*n* detectors is typically limited by generation/recombination (G-R) current which is characterized by an activation energy of approximately half the bandgap. This is confirmed by an activation energy of ~115 meV, obtained from the slope of *J_d_*/*T*^3^ vs. 1/*T* plot for T < 100 K, which is approximately half of the bandgap.

**Figure 4:**
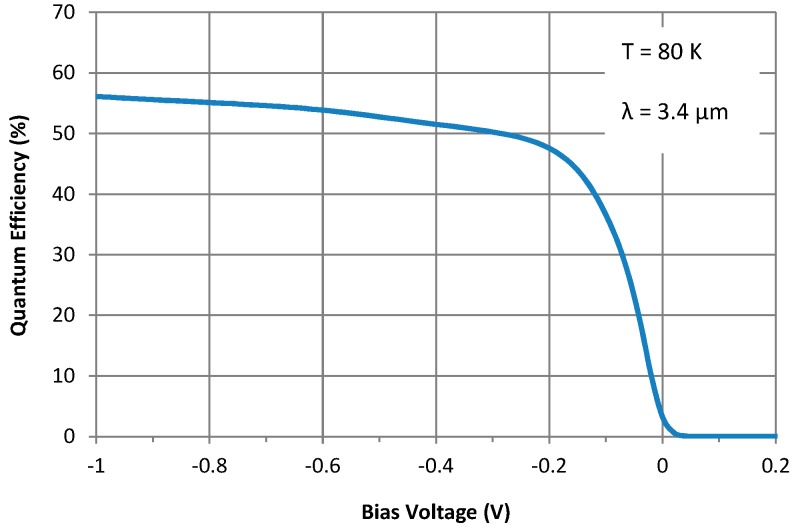
Variation of the external quantum efficiency (no AR coating) of the detector with bias at 80 K and 3.4 µm.

Photocurrent was measured using a calibrated blackbody and a set of notch filters at a few specific wavelengths. Then, the quantum efficiency was calculated through radiometric analysis. The resulting quantum efficiency of the detector and its variation with bias at 80 K and 3.4 µm are shown in Figure 4. It should be noted that this is the external quantum efficiency of the detector measured without using an antireflection (AR) coating. From Figure 4, it appears that the detector reaches 90% of max QE (i.e. the turn-on voltage) at a bias of ~ –0.2 V and QE increases slowly when the bias voltage magnitude is increased further. Unlike for a homojunction diode, *n*B*n* detectors are not expected to operate at 0 V, as there is no built-in field in the structure. However, pushing the operating bias voltage near 0 V is preferred, which can be done through optimization of the barrier band alignment and doping. The turn-on voltage at 80 K is reasonably small (200 mV), which decreases with increasing temperature [20].

**Figure 5:**
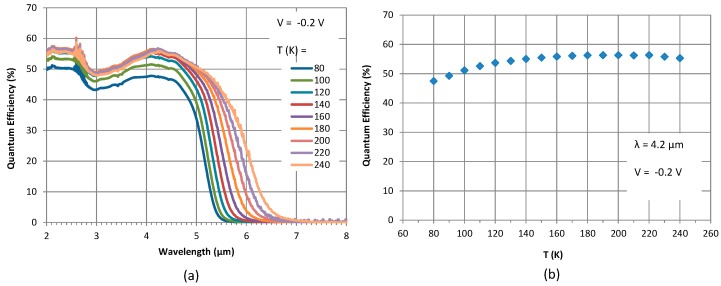
(**a**) Spectral quantum efficiency of the detector at –0.2 V at various temperatures and (**b**) variation of the peak quantum efficiency with temperature at 4.2 µm under –0.2 V.

Another important detector characteristic is the spectral response, which is typically measured using a spectrometer. An FTIR spectrometer was used to measure the relative spectral response of this device, which was scaled to spectral QE using the calibrated blackbody measurements. The spectral QE and its variation with temperature under a bias of −0.2 V is shown in Figure 5a. As observed, the cut-off wavelength (inflection point) at 80 K is approximately 5.25 µm (236 meV), which is extremely close to the designed bandgap of the superlattice (234 meV). If the 50% of peak QE is considered, the corresponding wavelength at the band edge is approximately 5.12 µm. This value will be considered as the cut-off for a comparison of the dark current against that of mercury cadmium telluride (MCT) detectors described by Rule 07 [29], as discussed in the next section. As the temperature is increased, the cut-off wavelength increases as expected, but the increase in peak QE is not ideal. As shown in Figure 5b, the magnitude of the peak QE (at 4.2 µm) increases with temperature in the 80–140 K range. At 140 K and beyond, QE saturates, indicating that the QE is likely absorption-limited at these temperatures. This value of QE will be compared with the maximum theoretical value, equal to the absorption efficiency, in the following section. The overall result indicates that this detector’s QE peaks at T > 140 K, making it suitable for HOT detectors.

## 4. Discussion

**Figure 6:**
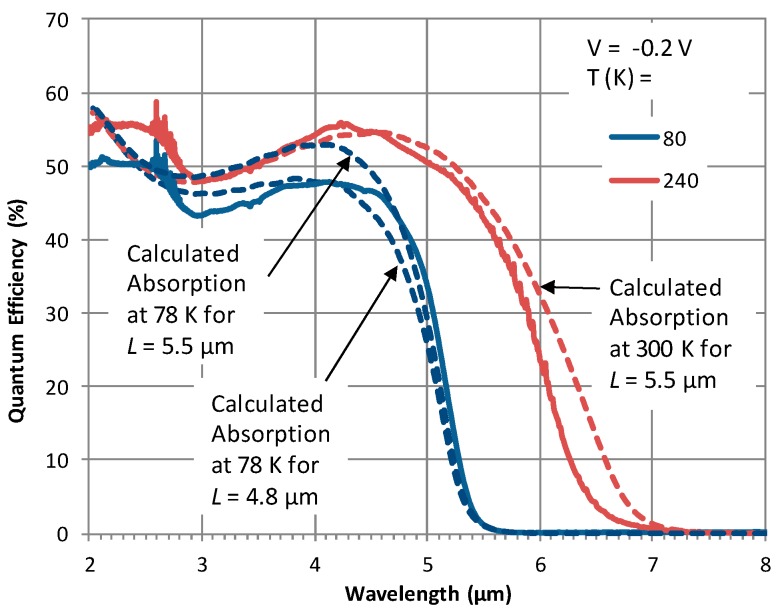
Comparison of the quantum efficiency measured at 80 and 240 K against the absorption efficiency calculated at 78 K (for *L* = 4.8 and 5.5 µm) and 300 K (for *L* = 5.5 µm), indicating a good agreement in the quantum efficiency values as well as the spectral shape. The highest temperature for quantum efficiency data available is 240 K and it was chosen to compare against the absorption at 300 K.

Using the experimentally determined absorption coefficient spectra shown in Figure 2, the total absorption in the 5.5 µm thick absorber was calculated [27] using the transfer matrix method under frontside illumination. This calculated absorption efficiency corresponds to the maximum theoretical quantum efficiency of the detector. A comparison of the absorption at 78 and 300 K is compared with the detector QE measured at 80 and 240 K, as shown in Figure 6. It was not possible to measure both absorption efficiency and the quantum efficiency at the same temperature, therefore, close values for the temperature were chosen for this comparison. It is clear that the value of QE at high temperatures is in good agreement with the absorption efficiency. The minor discrepancy observed in the overall spectral shape and the band edge could be due to two reasons: (i) the temperature difference (77 K vs. 80 K and 240 K vs. 300 K), impacting the bandgap and the cut-off wavelength, and (ii) optical resonant effects in the structure, which are very sensitive to the refractive index of the layers in the structure. Currently, the model considers real refractive index values reported in the literature, however, the actual values of the refractive index for the layers in the structure should be measured at corresponding temperatures and used in the model in order to further improve the simulated results.

Figure 6 also confirms that the maximum QE, shown in Figure 5b, is very close to the maximum theoretical QE, i.e. collection efficiency is near unity. However, for T < 140 K, the QE decreases as the temperature is decreased, indicating collection efficiency is less than 1 at these temperatures. Assuming that the diffusion length, *L*, is lower than the absorber thickness (= 5.5 µm), the absorption in a portion of the absorber with a thickness equal to *L* measured from the barrier/absorber interface, as indicated in Figure 1b, was calculated. Then, the value of *L* that gives the best fit between the absorption and QE was determined. At *T* ~80 K, this value was found to be ~4.8 µm. In other words, QE measured at 80 K corresponds to the collection of carriers generated within a ~4.8 µm region of the absorber. In Figure 6, the absorption efficiency spectra at 78 K correspond to *L* = 4.8 and 5.5 µm and the spectrum when *L* = 4.8 µm fits reasonably well with the quantum efficiency spectrum measured at 80 K. Moreover, when the temperature is increased from 80 to 140 K, *L* increases from 4.8 µm to 5.5 µm, respectively. While this is one straightforward way to explain the QE dependence on temperature, there could be other effects leading to the same observation such as variation in the barrier band alignment to the absorber with temperature and recombination of trapped holes at the interfaces [4].

As of today, MCT technology is still the leading technology for HOT detectors, while SLS technology has become a viable competitor. Therefore, it is worthwhile comparing the dark current between state-of-the-art MCT detectors described by Rule 07 [29] and the SLS detector reported in this paper. Defining the cut-off wavelength as the wavelength near the band edge corresponding to 50% of the peak QE (see Figure 5a), the cut-off wavelength values at different temperatures were determined and the Rule 07 dark current corresponding to those cut-off values and temperatures were calculated. It was then found that the dark current of this SLS detector is approximately a factor of 9, 4, and 3 higher than that of Rule 07 at 160, 180, and 200 K temperatures, respectively. 

The dark current and quantum efficiency of the detector reported in this paper are comparable to similar InAs/InAsSb SLS detectors recently reported in the literature. Ting et al. [4] have reported an InAs/InAsSb SLS *n*B*n* detector with a quantum efficiency of ~52% and dark current of 9.6 × 10^−5^ A/cm^2^ (a factor of ~4.5 higher than Rule 07) at ~157 K. With a detailed analysis of dark current characteristics, Rhiger et al. [30] have reported a similar InAs/InAsSb SLS *n*B*n* detector exhibiting a dark current 5 times higher than Rule 07. Furthermore, a comprehensive review of antimony-based detectors has been reported by Rogalski et al. [2] With this level of dark current performance and external quantum efficiency >50%, it can be predicted that SLS detectors are well within the reach of performance of InSb detectors but at high temperatures, promising as a candidate for HOT detectors.

**Figure 7:**
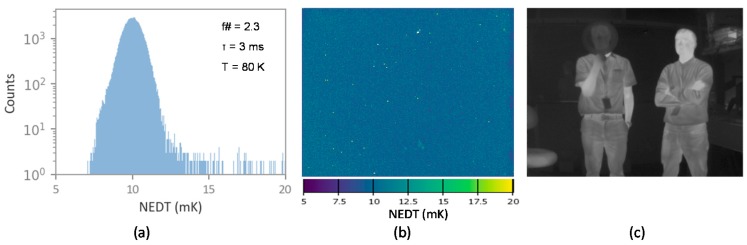
(**a**) Noise-equivalent temperature difference (NEDT) histogram of a 320 × 256 focal plane array (FPA) at 80 K; (**b**) NEDT operability map; and (**c**) an image taken at 80 K with f/2.3 optics and a 3 ms integration time.

To demonstrate the imaging performance, a 320 × 256 detector array with 30 µm pitch was fabricated, flip-chip bonded to a commercial readout integrated circuit chip (FLIR ISC9705), and tested to obtain performance metrics. As shown in Figure 7, the FPA exhibits promising results, including a median noise-equivalent temperature difference (NEDT) of 10 mK. This FPA also showed good uniformity and image quality up to about 140 K. Furthermore, these performance metrics agree with the characteristics measured at the single element detector level, discussed earlier in this paper.

## 5. Conclusions

A MWIR *n*B*n* detector designed using InAs/InAsSb SLS was reported. Detector characteristics were measured and analyzed with an emphasis on high temperature operation. At 160 K, this detector exhibits dark current of 9 times Rule 07 and peak quantum efficiency of 56% (~84% of internal quantum efficiency). The turn ON voltage is at or below −200 mV over the full temperature range. It was estimated that the diffusion length of the SLS is approximately 4.8 µm at 80 K, which increases to a value comparable to the absorber thickness (5.5 µm) when the temperature is increased to 140 K. Comparing the calculated absorption efficiency and the measured detector quantum efficiency, it was possible to conclude that the detector exhibits nearly 100% collection at temperatures higher than 140 K. While the performance metrics reported here do not meet those of InSb detectors yet, SLS technology continues to improve with the promise that it has the potential to deliver future HOT detectors required for many applications.
